# How Do They Really Arise?

**Published:** 1921-08-13

**Authors:** 


### ALIMENTARY DISORDERS OF INFANTS.

### How Do They Really Arise?

During the recent hot weather there will be few practitioners who did not encounter that troublesome condition incompletely classified as infantile diarrhœa and vomiting. From time to time a definite explanation of these outbreaks has apparently arrived, but, though often locally correct, none has ultimately proved applicable to all cases, and therefore most of us still tend to stand by whichever theory was most popular during our house-physician days. It is largely true that whatever treatment we then adopted with success will prove equally good to-day. Nevertheless, particularly from the preventive viewpoint, it is well to consider for a moment our exact powers in both understanding and handling this scourge to the infant life of Britain.

### The Bacterial Theory.

As a matter of fact, the rôle played by micro-organisms in the etiology of infantile vomiting and diarrhœa is still quite ill-defined. There are, indeed, two directly opposed schools of thought upon this point. On the one hand it is held that bacteria are of small importance and very rarely provide the determining cause. On the other they are held entirely responsible. As Dr. Frederick Langmead rightly points out, the confusion is increased by forgetting that diarrhœa and vomiting are, after all, only symptoms.

That excessive carbohydrate fermentation occurs and with obvious results is allowed by both sides. Also the facts that (*a*) these cases are much commoner in bottle-fed babies and in those of poor hygienic surroundings, and (*b*) are certainly more frequent during the summer months, provide the remainder of the common ground. The essential disagreement occurs concerning the part played by direct infection.

We find it hard, ourselves, to accept the statement that bacteria act merely as fermentative agents, or that they are, in the shape of any special strain, not proper to the particular local outbreak. It seems in practice that case series follow a very definitely infective line, and, to take a typical instance, we may quote that recorded in the *Lancet* last year by Sherwood.

He bases his comments upon the twenty-five years' records of a home where on an average seventeen infants from one month to two years old were lodged, fed, and clothed each year. For the first three years diarrhœa was seldom absent, and six deaths resulted from it. For the remaining twenty-two years no case of diarrhœa originated in the home, and no fatality from this cause was recorded. This change coincided exactly with the inauguration of a strict system of dealing with the children, and one can set down the main points as follows:

(1) The food was kept in a larder and, except at meal-times, was out of all contact with the children and their service. (2) A separate room was used for the changing of napkins, and, where possible, a separate nurse for the toilet. (3) The nates were washed and bathed with a disinfectant. (4) The nurses' and babies' hands were disinfected. (5) The napkins were immediately placed in a disinfectant.

It is true that there is nothing surprisingly novel in these five points, but nevertheless their success is remarkably illustrative. The larder is the obvious protection against germ-laden dust and flies. The separate room provides simply the same barrier which, in ordinary hygienic life, protects the adult. The disinfecting of hands and linen, if properly carried out, gives its obvious benefit. But the above was merely the ordinary routine, and we should also note what occurred in a suspected case. It is probably even more explanatory of the home's success.

When vomiting occurred (other than simple food regurgitation) the child was immediately removed to an isolation room. It was carefully washed, after all its clothes had been removed and placed in disinfectant. A complete set of clean clothes was then put on, and no milk given for several hours, the mouth being frequently washed out meanwhile with boracic solution. To those who have the handling of infectious disease, the sudden exclusion of the malady from this home should strongly suggest that bacteria decidedly do play a great part in the transference of infantile vomiting.

### Infection Derived from Adults.

So far, however, we have considered mainly child-to-child transference. The possibility of the adult as an infecting agent must not be forgotten. Dr. Langmead, in his summary in the current *Medical Annual*, gives us a case in point.

A child arrived at a certain home "in a pitiable condition due to gastro-intestinal disturbance, and was isolated and placed under the strict system of the institution. As it failed to benefit in the usual manner, all possible causes for the persistence of the trouble were investigated. It was found that the mother was continually kissing the child, and, when examined, she was found to have a mouth full of carious teeth. After this was prevented, and the teeth were attended to, the child made a rapid recovery." In short, the plea is for the application of surgical cleanliness in the nursery, because even if all cases of infantile diarrhœa and vomiting are not of bacterial origin, there seems very little doubt that a high percentage arise in this way.

### Non-bacterial Causes.

Nevertheless, there certainly remains a proportion of severe cases in which it is not only impossible to trace an infection, but also impossible to detect any abnormality in the intestinal organisms.

This has been proved by long series of clinic investigations (the details of which it is, in a short note, impossible to report). But their reader becomes quite prepared to accept the dictum that in medical practice we should definitely distinguish between functional and inflammatory (bacterial) forms. Admittedly the occurrence of both would in all probability increase during hot, exhausting weather.

In any case the non-assimilation of food, direct passage of undigested material, and a microscopical examination of the evacuated fœcal products will, together with simple bacteriology, serve to separate the two classes, and in this way the supporters of the bacterial and the non-bacterial cases will be satisfied. Both schools are almost certainly correct. There are many functional, fermentative cases, and many, probably more, cases due to inflammatory bacterial reaction. The simple symptoms in both types are practically the same; differentiation upon clinical signs is a matter of remarkable difficulty. The clinical laboratory is more likely to help us if we have time to seek its aid. But do not let us fall into the error of considering all these infantile cases as explained by anyone of opposing sets of theories. Bacteriological isolation and the handling of the child as a highly infectious case may be so much wasted energy. On the other hand, it may be just the reverse. As we have said, the moral is largely summed up in a plea for the aseptic nursery, but there is also the need for more habitual laboratory investigation before we can dogmatise on any of these alimentary disturbances.

